# Localized bullous pemphigoid masquerading as venous leg ulcers in a patient with chronic venous insufficiency

**DOI:** 10.1016/j.jdcr.2026.04.048

**Published:** 2026-04-30

**Authors:** Alexis Perry, Saman Namazian, Tony Vu, Joshua Mervis

**Affiliations:** a Tufts University School of Medicine, Boston, Massachusetts; b Department of Dermatology, Tufts Medical Center, Boston, Massachusetts

**Keywords:** bullous pemphigoid, BP230, BP180, dupilumab, immunobullous disease, localized bullous pemphigoid, venous insufficiency

## Introduction

Bullous pemphigoid (BP) is an autoimmune blistering disorder classically affecting older adults, characterized clinically by tense bullae and histologically by subepidermal blister formation with eosinophils and/or neutrophils. The pathophysiology is driven by autoantibodies against components of the hemidesmosome complex, most importantly BP180, a transmembrane protein. Autoantibodies against the intracellular protein BP230 are also often present, though their impact on disease development is less clear.^1^ Although BP commonly presents in a generalized distribution, localized variants occur rarely and can be diagnostically challenging.^2^ Localized BP, comprising ∼2.5% of reported cases, tends to occur at sites of trauma, scarring, radiation, and chronic inflammation.^3^^,^^4^ Chronic venous insufficiency (CVI) has been reported as a local trigger in 2 cases of localized BP.^5^^,^^6^ We present a rare case of localized BP initially mistaken for recurrent edema blisters and venous ulcers, successfully treated with dupilumab and compression. This underscores the potential diagnostic challenges of atypical BP presentations, and highlights the potential role of dupilumab, recently approved for the treatment of BP, in achieving sustained remission in the setting of localized BP triggered by CVI.

## Case Synopsis

A 76-year-old man with type 2 diabetes, atrial fibrillation, stage 2 chronic kidney disease, and long-standing CVI with history of venous leg ulcers presented with bilateral lower extremity swelling and ulcers of the left lower leg.

**Fig 1:**
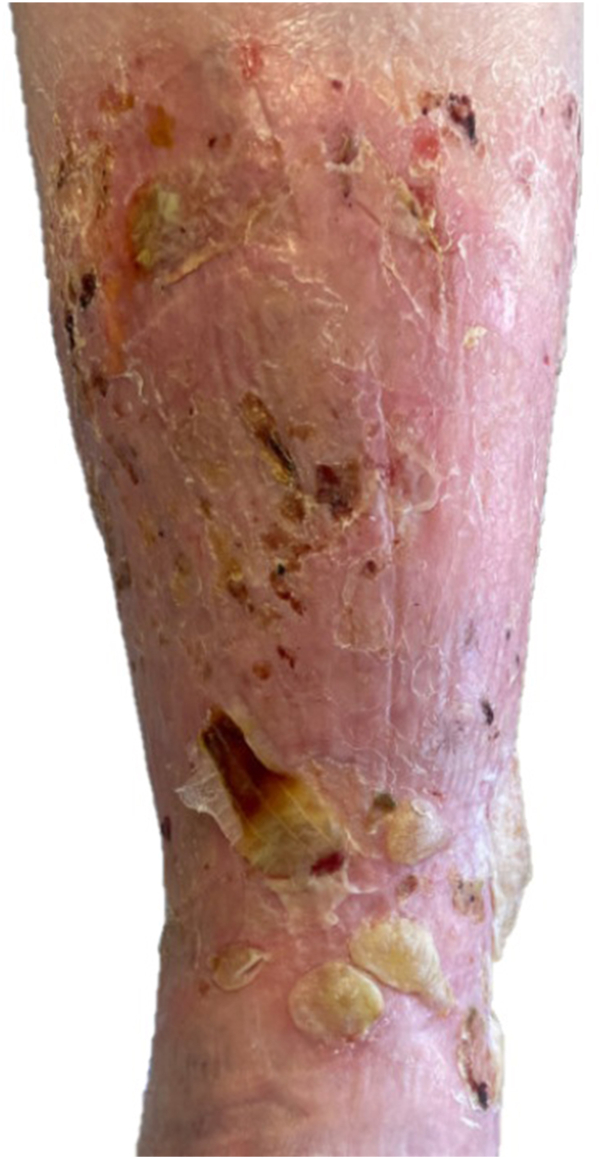
Anterior left lower extremity demonstrating localized tense bullae, shallow ulcers and erosions, moderate edema, and background scaly pink to hyperpigmented plaques.

Physical examination demonstrated bilateral lower extremity edema with multiple varicosities. The left anterior lower leg had several shallow ulcers and erosions with scattered tense bullae overlying pink to hyperpigmented scaly plaques (Fig 1). No bullae were identified elsewhere on the body. Venous duplex ultrasound revealed chronic occlusion of the left small saphenous vein without deep venous reflux. The patient had a history of venous leg ulcers and had previously healed with multilayer compression wraps and absorbent dressings. Similar treatment was reinitiated; however, despite edema control and trials of various dressing materials, the patient continued to develop new bullae and ulcers.

**Fig 2:**
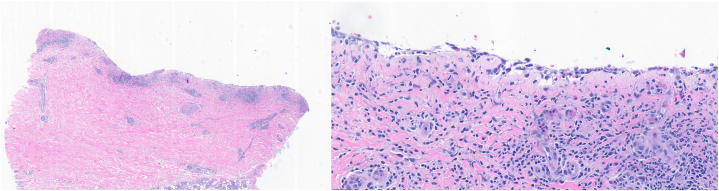
H&E stain of punch biopsy from a blister edge, demonstrating an ulcer with dermal fibrosis and a subepidermal split. Scattered thrombosed superficial vessels, proliferation of small blood vessels, and perivascular lymphohistiocytic infiltrate with abundant eosinophils are present. No bacteria, fungi or mycobacteria were detected with PAS, GMS, AFB, and Gram stains.

A punch biopsy from a blister edge was performed and revealed a subepidermal split with abundant eosinophils and a superficial perivascular lymphohistiocytic infiltrate (Fig 2), suggestive of BP. A second perilesional specimen was sent for direct immunofluorescence. However, it was nondiagnostic due to loss of the dermo-epidermal junction during processing. Serologies assessed via ELISA showed markedly elevated BP180 and BP230 autoantibodies at 60.3 and 107.9 U/mL, respectively (reference <9 U/mL). Taken together with the clinical presentation, these findings were consistent with a diagnosis of BP.

**Fig 3:**
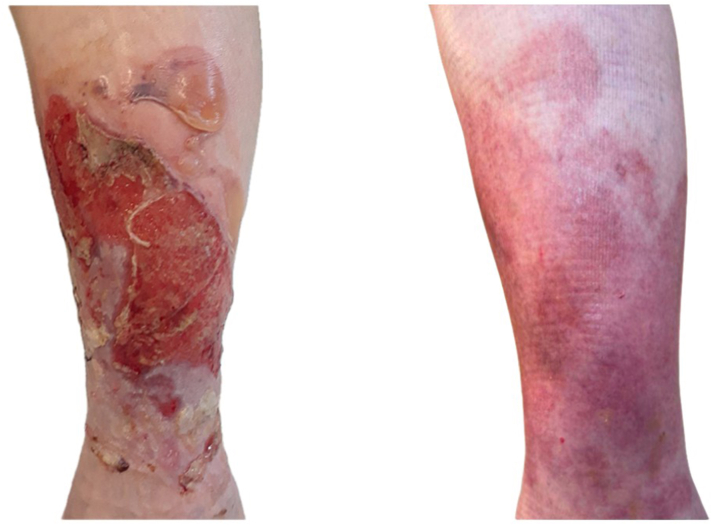
Left lower extremity with large ulcer and adjacent tense bullae prior to initiation of dupilumab (left) and after 2 months of therapy (right) with complete wound healing and resolution of bullae.

Initial treatment with multilayer compression wraps and clobetasol 0.05% ointment under occlusion yielded limited improvement. Therefore, systemic therapy with dupilumab was initiated with a 600 mg loading dose followed by 300 mg subcutaneously every 2 weeks. The patient had rapid clinical improvement with complete wound healing and cessation of blister formation after 2 months (Fig 3). Compression wraps were then transitioned to compression stockings with clobetasol application 3 times weekly for maintenance. An unintended 6-week interruption in dupilumab due to medication access issues led to localized relapse with recurrence of ulcers and bullae. Fortunately, complete resolution was achieved again following resumption of dupilumab in conjunction with compression.

## Discussion

Localized BP is a rare disease, and atypical presentations can be especially challenging to diagnose.^3^ This case report describes a patient with CVI and recurrent lower extremity wounds in which CVI acted as a local trigger for development of BP.

It has previously been hypothesized that CVI and associated blood extravasation may trigger the generation of localized BP by leading to immunologic dysregulation, including the generation of autoreactive T cells and the production of autoantibodies.^7^ Venous congestion may enhance local BP180/BP230 antibody activity and inflammatory signaling, precipitating blister formation; however, some previous reports of localized BP were negative for BP180 and/or BP230 antibodies.^3^^,^^8^

While localized BP can often be treated with topicals alone, our patient developed more severe localized disease that required systemic treatment. Dupilumab, an interleukin (IL)-4 receptor alpha subunit (IL-4Rα) antagonist blocking IL-4 and IL-13 signaling, has recently gained approval by the Food and Drug Administration for the treatment of BP. Given its safety and efficacy, dupilumab was considered a preferable long-term treatment option for our patient. In this case, dupilumab induced complete remission and provided rapid control after disease relapsed off treatment, highlighting its therapeutic potential beyond classic generalized disease and in instances of severe localized BP as well. One case report previously reported favorable treatment outcomes associated with dupilumab in a patient with radiotherapy-induced localized BP, the most commonly reported etiology of the localized variant.^9^ Treatment of CVI-induced localized BP with dupilumab has not been previously described.

Given the lower incidence compared to generalized disease, localized BP may easily be overlooked, especially in the setting of CVI where lower extremity edema blisters and venous leg ulcers often occur. When wounds do not respond as expected to presumed standard therapies, there must be consideration for alternative or atypical etiologies. In such cases, biopsy is warranted. Autoantibody serologies should be obtained when bullae are present and can be useful in making a diagnosis, particularly when biopsy results are not definitive. Nonetheless, autoantibody positivity will not be seen in all cases of localized BP.^3^^,^^8^ Although localized BP often follows a limited course, progression to generalized disease has been reported in up to 37% of cases, particularly among patients positive for BP180 autoantibodies.^10^ Furthermore, some reports suggest that despite higher anti-BP180 titers being associated with worse outcomes and severe refractory disease in patients with systemic BP, these patients maybe more sensitive to anti-IL-4 therapy such as dupilumab.^8^ Therefore, one can speculate about the potential for systemic therapies such as dupilumab to limit progression of localized BP to generalized disease, particularly in patients with BP180 autoantibody positivity.

## Conclusion

This case demonstrates an atypical presentation of localized BP mimicking edema blisters and venous leg ulcers and underscores the challenges of diagnosing localized BP in the setting of chronic venous disease. BP should be considered in patients with persistent localized blistering attributed to edema despite adequate compression therapy. Finally, this case highlights the potential utility of dupilumab in the treatment of localized BP.

## Conflicts of interest

None disclosed.
